# Book Reviews

**Published:** 1896-03

**Authors:** 


### Book Reviews.

MOULIN'S TREATISE ON SURGERY. Edited by John B. Hamilton, M. D., LL. D. Third American edition. Philadelphia: P. Blakiston, Son & Co., 1895.

The favorable reception of the previous editions of this work has induced the American editor to undertake to revise the treatise and to bring it up to date and thus render it more useful to the profession in the United States. New matter has been added and some of the subjects have been rewritten.

The work is particularly full on treatment and the advice given is, in general, practical and trustworthy.

It is somewhat surprising that the author is not inclined to advise electrolysis for the removal of nævi as it is clearly proven that this method yields better results than any other.

The chapter on appendicitis or, rather, typhlitis and perityphlitis is not up to the standard of recent exhaustive articles on the subject which have appeared in the medical press.

Altogether, the work can be recommended as an excellent guide to both students and practitioners.

---

AMERICAN TEXT-BOOK OF SURGERY, FOR PRACTITIONERS AND STUDENTS.—Edited by William W. Keen, M. D., LL. D., and J. William White, M. D., Ph. D. Second edition, carefully revised. Philadelphia: W. B. Saunders, 1895. Royal 8vo: pp. 1,248. Cloth, $7.00; sheep, $8.00; half russia, $9.00. (For sale by publisher by subscription only.)

This book is fully up to the standard of its predecessors in the "American Text-Book" series. Every department of surgery is fully covered and the text explained at every step by illustration. The authors are all selected from special fitness and have succeeded in getting together a work that is unsurpassed of its kind.

All that is obsolete has been cut out. So that, as a text-book, it may be relied upon as presenting nothing that is not in accord with our present knowledge and, hence, safe for the student to learn.

A HAND-BOOK OF OBSTETRIC NURSING, FOR NURSES, STUDENTS AND MOTHERS. Comprising the course of instruction in obstetric nursing given to the pupils of the Training School for Nurses connected with the Woman's Hospital of Philadelphia. By Anna M. Fullerton, M. D. Fourth revised edition. Illustrated. Philadelphia: P. Blakiston, Son & Co., 1012 Walnut St., 1895.

The steady decline in the number of cases of puerperal sepsis, one of the greatest dangers that assails the parturient woman is due entirely to a practical recognition of the "value of cleanliness, antisepsis and eternal vigilance" on the part of the physician and nurse. This little manual is designed specially for the use of the latter, and explains clearly and practically the conditions with which she is likely to be confronted. The importance to the nurse of a thorough understanding of the dangers of the puerperium cannot be overestimated, as it keeps her constantly on the alert and prevents carelessness and inattention to cleanliness. There are added some chapters on the care of the newborn infant and some of the common ailments to which it is subject. The price of the book is $1.00.

---

PRINCIPLES OF SURGERY. By N. Senn, M. D., Ph. D., LL. D., Professor of Practice of Surgery and Clinical Surgery in Rush Medical College, Chicago; Professor of Surgery in the Chicago Polyclinic; Attending Surgeon to the Presbyterian Hospital; Surgeon-in-Chief to St. Joseph's Hospital; Ex-President American Surgical Association, etc. Second edition; thoroughly revised. Illustrated with 178 wood engravings and five colored plates. Royal octavo; pp. xvi, 656. Extra cloth, $4,50 net; sheep or half russia, $5.50 net. Philadelphia: The F. A. Davis Co., Publishers, 1914 and 1916 Cherry street.

The merit of Senn's Principles of Surgery is further attested by the necessity from demands on the publisher to issue a new and revised edition. We know of no more valuable work, than this upon the subject, as the author combines the qualities of a profound pathologist with all the technical skill of a practical surgeon of wide experience and much talent. We have no doubt but that the second edition will meet with the ready acceptance which is due it.

